# The Status of Neuroimaging with SPECT and PET in Germany: Results from the 1 ^st^ Survey on Nuclear Neuroimaging in Germany in 2023

**DOI:** 10.1055/a-2566-1487

**Published:** 2025-04-15

**Authors:** Ralph Buchert, Alexander Drzezga, Mathias Schreckenberger, Karl Josef Langen, Philipp T Meyer, for the Working Group Nuclear Neuroimaging of the German Society of Nuclear Medicine (DGN e.V.)

**Affiliations:** 1 Department of Diagnostic and Interventional Radiology and Nuclear Medicine, University Medical Center Hamburg-Eppendorf, Hamburg, Germany; 2 Institute of Neuroscience and Medicine (INM-2), Forschungszentrum Jülich, Jülich, Germany; 3 Department of Nuclear Medicine, Faculty of Medicine and University Hospital Cologne, University of Cologne, Cologne, Germany; 4 German Center for Neurodegenerative Diseases (DZNE), Bonn-Cologne, Germany; 5 Department of Nuclear Medicine, Johannes Gutenberg University, Mainz, Germany; 6 Institute of Neuroscience and Medicine (INM-3/INM-4/INM-5/INM-11), Forschungszentrum Jülich, Jülich, Germany; 7 Department of Nuclear Medicine, University Hospital RWTH Aachen, Aachen, Germany; 8 Center of Integrated Oncology, Aachen Bonn Cologne Düsseldorf, Germany; 9 Department of Nuclear Medicine, Medical Center – University of Freiburg, Freiburg, Germany

**Keywords:** nuclear imaging, brain, SPECT, PET, Germany, survey

## Abstract

The advent of disease-modifying therapies for neurodegenerative diseases may result in a growing demand for nuclear neuroimaging procedures presenting opportunities but also challenges to the nuclear medicine community. Whether capacity and expertise in Germany are sufficient to meet an increasing demand for nuclear neuroimaging is under discussion. Against this background, the Neuroimaging Working Group of the German Society of Nuclear Medicine initiated the first survey on the status of nuclear neuroimaging in Germany in 2023. 82 institutions participated in the survey: 33 practices, 15 community hospitals, 34 university hospitals. Primary findings were the following. In practices, brain scans are less frequently performed than in hospitals and are often limited to dopamine transporter SPECT. Brain PET is mainly performed in hospitals, and in community hospitals it is often restricted to FDG PET. Nevertheless, availability of amyloid PET with well-certified quality can be taken for granted. Thus, access to amyloid PET will not be a major bottleneck for new treatments of Alzheimer’s disease. Adequate reimbursement and clear anchoring in clinical guidelines have the greatest potential to advance nuclear neuroimaging in Germany. Clinical dopamine transporter SPECT is largely in agreement with procedure guidelines. An area for improvement is the limited availability of MR images to avoid misinterpretation of structural/vascular lesions as nigrostriatal degeneration. The survey provides the first systematic assessment of the status of nuclear neuroimaging in Germany. It underscores the capacity of the German nuclear medicine community to meet an increasing demand for neuroimaging procedures, its adherence to procedure guidelines and identifies topics for improvement.

## Introduction

The development of disease-modifying therapies holds promise to become a major breakthrough in the treatment of neurodegenerative diseases, especially Alzheimer’s disease (AD) 1 , but also Parkinsonʼs disease 2 3 . In addition, the role of nuclear neuroimaging procedures in clinical guidelines has been strengthened by the aggregation of evidence 4 5 . These developments present not only opportunities but also challenges to the nuclear medicine community 6 7 8 . In particular, the necessity of widespread availability of nuclear neuroimaging procedures is a recurring topic of discussion during the development of clinical guidelines.

Against this background, the Nuclear Neuroimaging Working Group of the German Society of Nuclear Medicine (Deutsche Gesellschaft für Nuklearmedizin e.V., DGN) initiated the first survey on the status of nuclear neuroimaging in Germany in 2023. This manuscript reports the results of the full survey. Results specifically on the capacity for amyloid PET in Germany have been reported previously 9 .

## Materials and Methods

Nuclear medicine physicians were contacted via email using the email lists of the DGN and the professional organization of German nuclear medicine physicians (Berufsverband Deutscher Nuklearmediziner e.V., BDN). A questionnaire was provided as a MS Excel document attached to the email as well as for download from a link specified in the text body of the email. The emails were first sent on October 06 and October 09, 2023. Participants were asked to send the completed questionnaire per email to a central address specifically set up for this purpose ( UmfrageNeuronuk2023@uke.de ). The deadline was set to October 31, 2023. Reminder emails were sent on November 15 and November 17, 2023 using the same DGN and BDN email lists. The deadline was extended to December 10, 2023. Finally, nuclear medicine departments at German university hospitals that had not responded yet were contacted by individual emails during January 2024.

The questionnaire consisted of 33 questions grouped into 3 parts: part A “general information” (20 questions), part B “expertise & acceptance” (7 questions), and part C “dopamine transporter SPECT methods” (6 questions). In order to limit the time required to complete the questionnaire to a maximum of 10 minutes, predefined response options were provided and participants were asked to respond by ticking one (or several) of the options. This also applied to the questions on the frequency of certain imaging procedures, for which different ranges were provided as response options (e.g., none, 1–25, 26–100, 101–200, 201–500, >500). The participants were asked to select the appropriate range “off the top of their head” (i.e., without searching databases). None of the questions required free text answers.

Imaging procedures addressed by the survey included non-brain imaging procedures performed for neurological indications such as cisternography and [ ^123^ I]MIBG scintigraphy of the cardiac sympathetic innervation to support the differential diagnosis of neurodegenerative parkinsonian syndromes. In the following, these are subsumed under “neuroimaging procedures” in order to simplify the notation.

The results are presented separately for practices (PR), non-university community hospitals (CH) and university hospitals (UH). No distinction was made between medical supply centres (Medizinische Versorgungszentren, MVZ) associated with an UH and the corresponding UH. In particular, it was assumed that estimates on the frequency of a given imaging procedure provided by an UH with MVZ represented the total number of examinations performed by the UH and the associated MVZ combined. Single MVZ (not associated with an UH) were assigned to the PR category 10 .

“Estimated” mean frequencies (e.g., of a certain imaging procedure) across responding institutions were computed by representing the predefined ranges by their mean value. For example, the range “1–25” was represented by (1+25)/2 = 13. The highest range was represented by 50% of the width of the second highest range above the cutoff. For example, the highest range “>500” after the second highest range “201–500” was represented by 500+(500–201+1)/2 = 650.

The association between two ordinal variables was tested by the Goodman-Kruskal gamma test. The threshold for statistical significance was set at two-sided p < 0.05.

## Results & Discussion

A total of 85 completed questionnaires were received. Two questionnaires were excluded because they were copies (submitted by different persons from the same institution), one questionnaire was excluded because it was submitted from a non-german institution. The remaining 82 questionnaires were included.

Thirty-three (40%) of the 82 included questionnaires were from PR, 15 (18%) from CH and 34 (42%) from UH. The distribution of the responding institutions across the German federal states is given in **Supplementary Table 1** .

### Total number of nuclear neuroimaging procedures represented by the survey

**Fig. 1:**
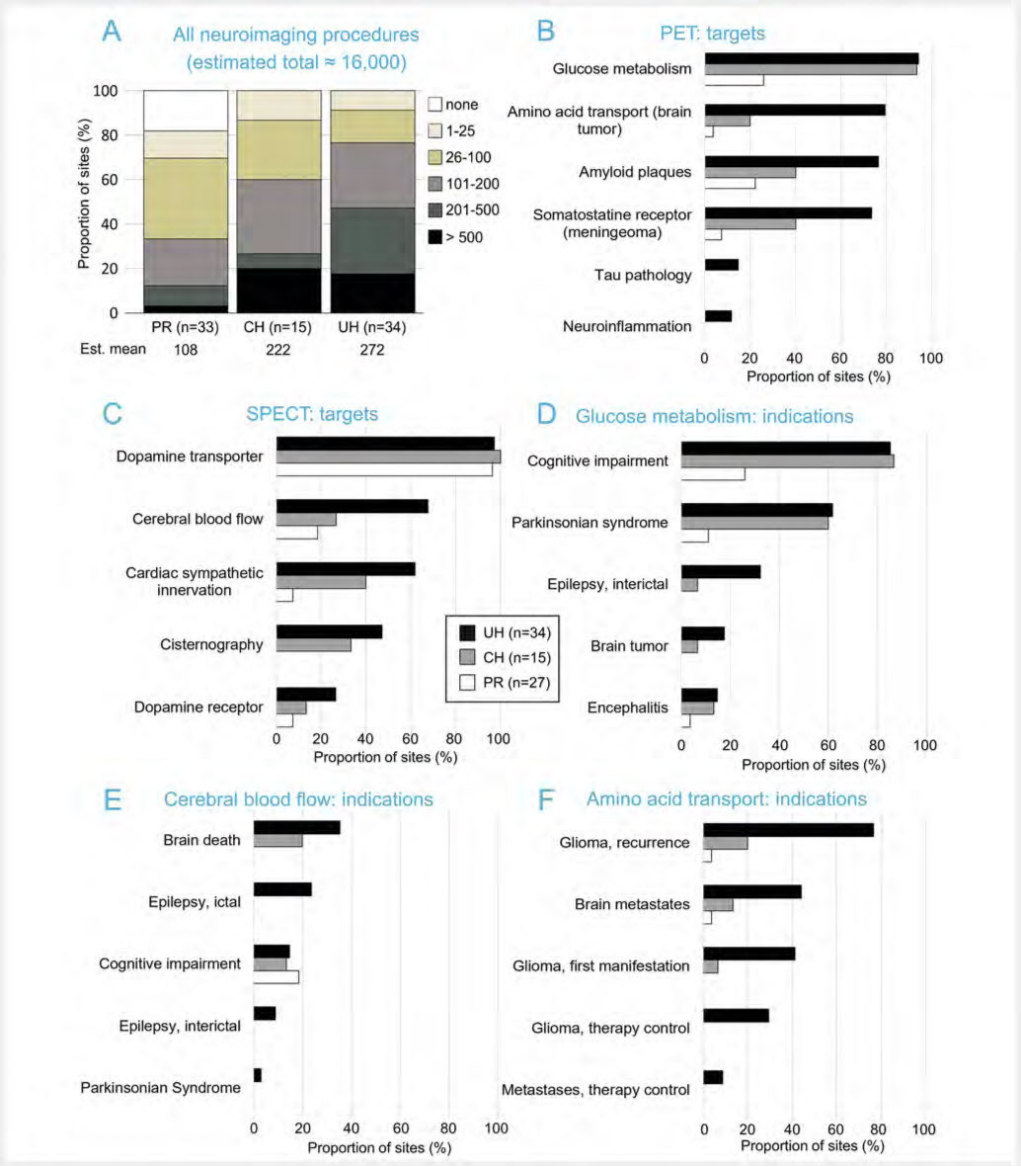
Total number of nuclear neuroimaging procedures, their targets and indications. **A:** Total number of nuclear neuroimaging procedures currently performed per year at the responding institutions independent of modality (SPECT, PET), target and indication. Six (18%) of the 33 PR reported to perform no nuclear neuroimaging at all. These PR were excluded from parts B-F of the figure, leaving 27 PR. **B, D, F:** PET targets ( **B** ) and most common indications for brain PET of regional cerebral glucose metabolism with [ ^18^ F]FDG ( **D** ) and for brain PET of regional amino acid transport with any amino acid tracer ( **F** ). **C, E:** SPECT targets ( **C** ) and most common indications for brain SPECT of regional cerebral blood flow ( **E** ). The institutions were asked to tick all targets currently examined independent of their frequency (multiple answers possible) and all indications that accounted for at least 10% of the corresponding imaging procedure (multiple answers possible). (PR = practice or medical supply center, CH = non-university community hospital, UH = university hospital).

The estimated mean number of neuroimaging procedures including both modalities (SPECT, PET), all targets and all indications was 108, 222 and 272 per year for PR, CH and UH, respectively ( Fig. 1 A). The estimated total number of neuroimaging exams including both modalities, all targets and indications across all responding institutions was approximately 16,000 per year.

### Targets and indications for nuclear neuroimaging

Targets and indications for nuclear neuroimaging are summarized in Fig. 1 **B–F** . Brain FDG PET for the detection (or exclusion) of altered cerebral glucose metabolism was performed at the vast majority (≥ 93%) of CH and UH, but only at about one quarter of the responding PR ( Fig. 1 B). The etiological diagnosis of clinically uncertain cognitive impairment was the most frequent indication for brain FDG PET, closely followed by the differential diagnosis of neurodegenerative parkinsonian syndromes ( Fig. 1 D). Amyloid PET, somatostatine receptor PET (meningioma) and amino acid PET were also performed at the majority of UH ( Fig. 1 B). The most common indication for amino acid PET was suspected glioma recurrence but also diagnosis of recurrent brain metastases is becoming increasingly important ( Fig. 1 F).

In 2023, tau PET was performed in clinical practice in 5 of 34 UH (15%; Fig. 1 B). None of the PR and none of the CH performed clinical tau PET in 2023. This may be explained by the fact that approval of the first tau PET tracer, [ ^18^ F]Flortaucipir (Tauvid, Eli Lilly), by the European Medicines Agency (EMA) was only in August 2024 ( www.ema.europa.eu/en/medicines/human/EPAR/tauvid#authorisation-details ). At the time of writing (02/2025), [ ^18^ F]Flortaucipir was not yet commercially available in Germany. Furthermore, the version of the S3 guideline on dementia 4 valid until end of February 2025 only referred to cerebrospinal fluid testing for the assessment of the tau status in patients with suspected AD. Tau PET was not mentioned at all in this version. However, in the update of the S3 guideline end of February 2025, a recommendation was added to use tau-PET for the detection or exclusion of advanced AD-typical tau pathology in patients with uncertainty regarding the underlying disease after clinical and neuropsychological examination and possibly amyloid-PET.

Dopamine transporter (DAT) SPECT was performed at the vast majority (≥ 96%) of the responding institutions independent of their type (PR, CH, UH; Fig. 1 C). Brain perfusion SPECT was mainly performed at UH ( Fig. 1 C) and mainly for brain death diagnosis and presurgical evaluation of epilepsy patients ( Fig. 1 E). Interestingly, *ictal* brain perfusion SPECT was more widely used than *interictal* brain perfusion SPECT for the latter indication ( Fig. 1 E), despite the fact that ictal SPECT is much more demanding due to the unpredictable waiting period until the next seizure. This finding is in line with a recent study demonstrating that starting brain perfusion SPECT in the presurgical evaluation of epilepsy with an interictal scan to skip the ictal scan in case of a high-confidence interictal candidate for the seizure onset zone is not a useful approach 11 . In contrast, starting with an ictal scan to skip the interictal scan in case of a high-confidence ictal candidate for the seizure onset zone can be recommended 11 . Furthermore, most UH prefer FDG PET for interictal-only imaging in epilepsy patients ( Fig. 1 D). This suggests that interictal brain perfusion SPECT is mainly performed in combination with ictal SPECT in order to improve sensitivity and specificity for the identification of the seizure onset zone (e.g., by subtraction analyses).

### Frequencies of specific neuroimaging procedures

**Fig. 2:**
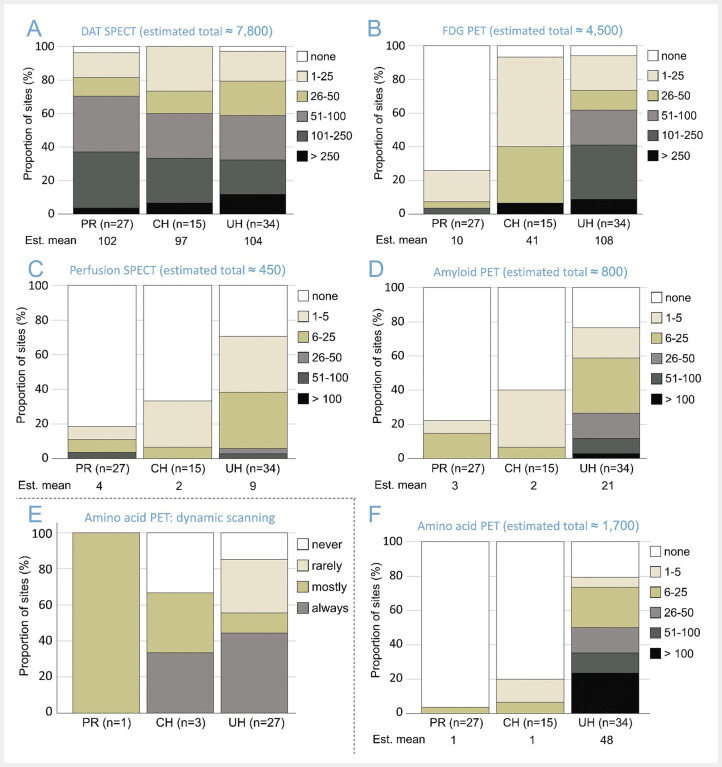
Annual number of specific neuroimaging procedures using SPECT (A, C) or PET (B, D, F). **A:** dopamine transporter (DAT) SPECT, **C:** brain perfusion SPECT. **B:** [ ^18^ F]FDG PET, **D:** amyloid PET, and **F:** amino acid PET. The 6 PR that reported to perform no nuclear neuroimaging at all were not included leaving 27 PR. Thus, percentages were computed relative to 27 PR, 15 CH or 34 UH. **E:** Proportion of institutions that reported to perform amino acid PET using a dynamic acquisition protocol with start at tracer injection (e.g. 0–40 min p.i. or 0–50 min p.i.). Percentages were computed relative to the number of PR (n=1), CH (n=3) and UH (n=27) that responded to this specific question. (PR = practice or medical supply center, CH = non-university community hospital, UH = university hospital).

The frequencies of specific neuroimaging procedures at the responding institutions are summarized in Fig. 2 .

DAT SPECT was performed with similar frequency, on average about 100 scans per year, at all responding institutions independent of their type ( Fig. 2 A). All other neuroimaging procedures were much more frequent at UH compared to both CH and PR with one notable exception: the responding CH performed on average 41 FDG PET per year, almost 40% of the annual number of FDG PET at the UH (108, Fig. 2 B). Thus, FDG PET clearly dominated the brain PET procedures at CH ( Fig. 2 **B, D, F** ).

The relatively high proportion of the responding PR not performing FDG PET (74%) and not performing amyloid-PET (78%) is largely explained by the fact that 19 (70%) of the 27 PR performing nuclear neuroimaging procedures had no PET scanner available. The proportion of responding CH and UH without PET was 7% and 3%, respectively. Among the PR with PET scanner, the frequency of FDG PET and amyloid PET was similar as in the CH with PET scanner ( **Supplementary Figure 1** ).

The estimated total annual number across all responding institutions was about 7,800 for DAT SPECT, 450 for brain perfusion SPECT, 4,500 for FDG PET, 800 for amyloid PET and 1,700 for amino acid PET. The dominant roles of DAT SPECT and FDG PET fit their high acceptance and level of recommendation for differential diagnosis and prognosis of parkinsonian syndromes and cognitive impairment in current national guidelines 4 5 . The higher frequency of DAT SPECT compared to FDG PET is probably due not only to the higher availability of SPECT compared to PET, but also to the fact that DAT SPECT is reimbursed by statutory health insurance, in contrast to FDG PET. Thus, assuming that the vast majority of the FDG PET scans represented by this survey most likely were not paid by statutory health insurance, their large number (≈ 4,500 per year) documents the importance of brain FDG PET for patient care, which should encourage health insurers to reconsider their reimbursement policies regarding brain FDG PET.

Interestingly, the majority (58%) of the institutions performing amino acid PET “mostly” or “always” used a dynamic acquisition protocol starting with tracer injection, probably mostly [ ^18^ F]FET ( Fig. 2 E). This is despite the fact that the diagnostic impact of additional dynamic imaging in [ ^18^ F]FET PET is controversial 12 .

### Representativeness of the survey

The number of 82 eligible questionnaires from the 1 ^st^ survey on nuclear neuroimaging in Germany is less than a third of the total number of 278 institutions that participated in the 9 ^th^ survey on myocardial perfusion SPECT in Germany in 2021 10 . In the latter, 131 PR, 58 CH and 29 UH reported to perform myocardial perfusion SPECT. Thus, the number of PR that reported to perform the nuclear imaging procedures of interest (myocardial perfusion SPECT or nuclear neuroimaging) was almost 5 times larger in the myocardial perfusion SPECT survey than in the nuclear neuroimaging survey (131/27 = 4.9). In contrast, the number of responding UH was larger in the neuroimaging survey compared to the myocardial perfusion SPECT survey (34 versus 29), indicating that the coverage of the German UH was slightly more complete in the neuroimaging survey. The association of German University Hospitals (Verband der Universitätsklinika Deutschlands e.V.) represents 38 full members and 5 associate members at the time of writing (02/2025).

On request, the National Association of Statutory Health Insurance Physicians (Kassenärztliche Bundesvereinigung) reported a total number of 12,400 outpatient DAT SPECT in 2022 (fee schedule item 40538). Assuming (i) that the frequency of DAT SPECT does not differ between privately and statutorily insured patients and (ii) that the proportion of privately insured patients in Germany is 11.2% 10 , the number of outpatient DAT SPECT in privately insured patients in 2022 is estimated to 1,560. Based on the number of 983 in-patient brain SPECT examinations in 2022 (OPS 3–720, all tracers) and assuming 95% DAT SPECT among these examinations (present survey), the number of in-patient DAT SPECT in 2022 is estimated to 930. Taken together, these numbers amount to about 14,890 DAT SPECT in 2022 in Germany (12,400 + 1,560 + 930). Thus, the estimated total of 7,800 DAT SPECT per year across all responding institutions of the current survey represents about 52% of all expected DAT SPECT examinations in Germany. This suggests that the 1 ^st^ survey on nuclear neuroimaging is as representative of nuclear neuroimaging in Germany as the 9 ^th^ survey is representative of myocardial perfusion SPECT, despite the considerably smaller number of responding institutions. The 9 ^th^ survey on myocardial perfusion SPECT recorded 54% of all myocardial perfusion SPECT examinations in Germany 10 .

### Amyloid PET of the brain

The development of anti-amyloid therapies for the treatment of AD 1 may result in a rapidly growing demand for amyloid PET 6 7 8 . For instance, based on a recent report of Canada’s Drug Agency on the readiness of the Canadian health care system for the introduction of anti-amyloid AD therapies 13 , the demand for amyloid PET in Germany may rise to almost 20,000 scans in the first year after approval of an anti-amyloid therapy. Whether availability, capacity and expertise for amyloid PET in Germany is sufficient to meet such a demand is still under discussion 9 .

In terms of general instrumentation and current workload, a recent survey by Fendler and Holzgreve 14 (approximately 180 PET scanners, on average providing 1,000 scans per year) suggests that there is PET scanner capacity in Germany for approximately 180,000 additional PET scans per year (assuming a reasonable 2,000 scans per scanner and year). Three additional requirements need to be met to be able to respond quickly to a growing demand for amyloid PET: (i) a sufficient number of formally trained physicians, (ii) practical experience in performing amyloid PET scans and (iii) good availability of amyloid tracers.

**Fig. 3:**
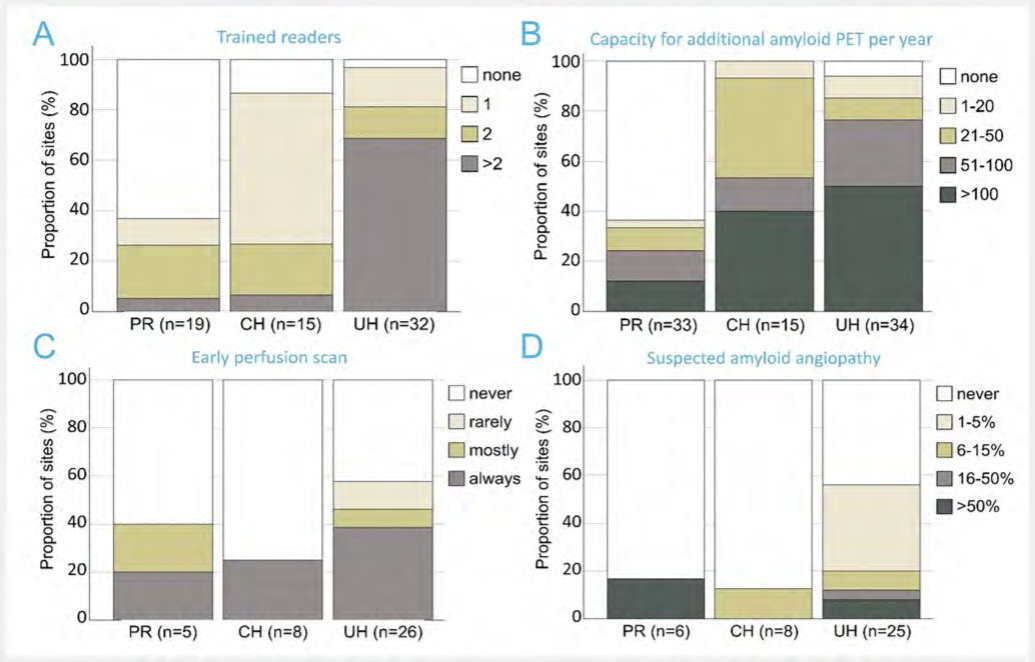
Amyloid PET. **A** : number of physicians at the institution that are certified to perform amyloid PET scans with one of the approved tracers (“reader training”), **B** : capacity to increase the number of amyloid PET scans per year, e.g. with the introduction of new therapies, assuming adequate reimbursement, **C** : early scanning immediately after tracer injection to assess regional cerebral blood flow (as a substitute for FDG PET), and **D:** relative frequency of suspected amyloid angiopathy as indication. The number of responding institutions differed between the questions and therefore is given separately for each subplot. (PR = practice or medical supply center, CH = non-university community hospital, UH = university hospital).

Data obtained from the manufacturers of [ ^18^ F]florbetaben (NeuraCeq, Life Molecular Imaging) and [ ^18^ F]flutemetamol (Vizamyl, GE HealthCare) via the German Electrical and Electronic Manufacturersʼ Association (ZVEI) indicate that ≥ 400 physicians in Germany are trained in amyloid PET with one or both of these tracers, with adequate coverage of all German federal states ( **Supplementary Figure 2** ). Among the UH that participated in the survey, 97% reported at least one trained reader, 69% reported three or more trained readers ( Fig. 3 A). While at least one trained reader was also available at most responding CH (87%), this fraction was considerably lower (37%) among the responding PR (in line with only few PR performing amyloid PET; Fig. 2 D).

The reported capacity for additional amyloid PET brain scans was more than 50 per year (1 per week) in 24%, 53%, and 76% of the responding PR, CH, and UH, respectively ( Fig. 3 B), amounting to approximately 5,000 additional scans per year in total. This is approximately six times the total number of clinical amyloid PET scans currently performed at the responding institutions (≈ 800, Fig. 2 D) and well comparable to the total number of [ ^18^ F]FDG PET brain scans they perform annually (≈ 4,500, Fig. 2 B). Assuming that the survey covered about half of the nuclear neuroimaging services in Germany (see “Representativeness of the survey”), the actual capacity for additional amyloid PET may be considerably higher, up to 10,000 amyloid scans per year.

Sufficient coverage with commercially available amyloid tracers may be assumed, as confirmed by the manufacturers and demonstrated by current multicentre amyloid PET imaging studies, such as the German coverage with evidence development study ENABLE 15 .

Taken together, these data suggest that the general conditions for increasing the number of amyloid PET scans are already in place in Germany. However, it is desirable that more PR and CH offer clinical amyloid PET to broaden the base for this test.

Remarkably, about 40% of all institutions that reported to currently perform amyloid PET acquire an early scan immediately after tracer injection as a surrogate of regional cerebral blood flow, although this is not an approved application ( Fig. 3 C). Since all available amyloid tracers show a sufficiently high single pass extraction in the brain for reliable detection of relevant regional *hypo* perfusion (though not necessarily *hyper* perfusion) 16 , early perfusion-phase imaging can support the differentiation between different AD subtypes 17 in amyloid-positive cases as well as the detection (or exclusion) and differentiation of non-AD neurodegenerative diseases in amyloid-negative cases 18 19 .

For further discussion, the interested reader is referred to our recent statement on the capacity of amyloid PET in Germany 9 .

Finally, it may be worth noting that the recent update of the German S3 guideline on dementia (February 28, 2025) added a recommendation not to use (approved) blood-based biomarkers alone for the diagnosis of cerebral amyloid pathology or other neuropathological aspects of neurodegenerative diseases.

### Expertise, acceptance and perceived frequency of neuroimaging procedures

**Fig. 4:**
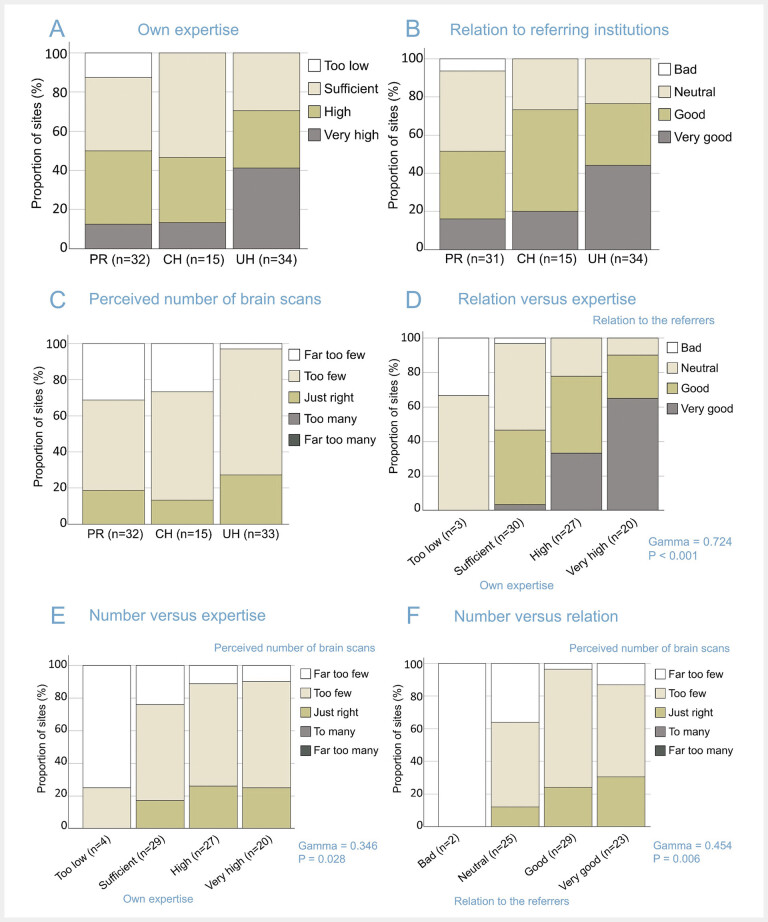
Expertise, acceptance and perceived frequency of neuroimaging procedures (A–C), and association between them (D–F): **A:** level of nuclear neuroimaging expertise in the institution (self-assessment), **B:** relationship with referrers for nuclear neuroimaging (self-assessment), **C:** perceived frequency of nuclear neuroimaging procedures. **D–F:** association between the level of nuclear neuroimaging expertise in the institution and the relationship with referrers for nuclear neuroimaging procedures ( **D** ) or with the perceived frequency of nuclear neuroimaging procedures ( **E** ) and between the latter two ( **F** ).

The results of the self-assessment of the responding institutions regarding their own level of expertise in nuclear neuroimaging, their relationship with referrers for neuroimaging, and their perception of the frequency of neuroimaging procedures in their institution are summarised in Fig. 4 .

The proportion of institutions that estimated their neuroimaging expertise as “high” or “very high” was larger among UH (71%) compared with CH (47%) and PR (50%; Fig. 4 A). However, even among UH almost one third (29%) reported only “sufficient” neuroimaging expertise. Approximately 10% of the responding PR considered their expertise “too low”. The relationship with referrers was considered “good” or “very good” by about 75% of the responding CH and UH ( Fig. 4 B). This proportion was somewhat smaller among PR (52%). Two (6%) of 31 PR estimated their relationship with the referring institutions as “bad”. The number of neuroimaging procedures performed at their institution was considered “too few” or “far too few” by the majority of the responding institutions more or less independent of their type (PR: 81%, CH: 87%, UH: 73%; Fig. 4 C). None of the responding institutions rated the number of neuroimaging procedures as “too many” or “far too many”.

Expertise, relationship with referrers and the perceived frequency of neuroimaging procedures were significantly associated pairwise ( Fig. 4 **D–F** ). The association was most pronounced between expertise and relationship (gamma = 0.724, p < 0.001; Fig. 4 D): the proportion of responding institutions with “good” or “very good” relationship with the referrers for nuclear neuroimaging increased from 0% for nuclear medicine institutions with “too low” expertise to 47% at “sufficient expertise”, 78% at “high” expertise, and 90% at “very high” expertise ( Fig. 4 D). This suggests that training of nuclear medicine physicians and technicians specifically in nuclear neuroimaging may greatly improve the relation between nuclear medicine facilities and referrers for nuclear neuroimaging. The practical relevance arises from the fact that a good relationship with (potential) referrers is one of the prerequisites to advance nuclear neuroimaging.

It should be noted that the results of the current survey on expertise, acceptance and perceived frequency of neuroimaging procedures represent the institutions that responded to the survey and most likely cannot be extrapolated without modification to non-responding institutions. We hypothesize that the proportion of institutions that do not currently perform neuroimaging will be higher among non-responding institutions than among the responding institutions, and that the level of expertise in nuclear neuroimaging is lower among institutions that do not perform nuclear neuroimaging procedures.

### Reasons for a too low number of neuroimaging procedures and measures to improve the acceptance

**Fig. 5:**
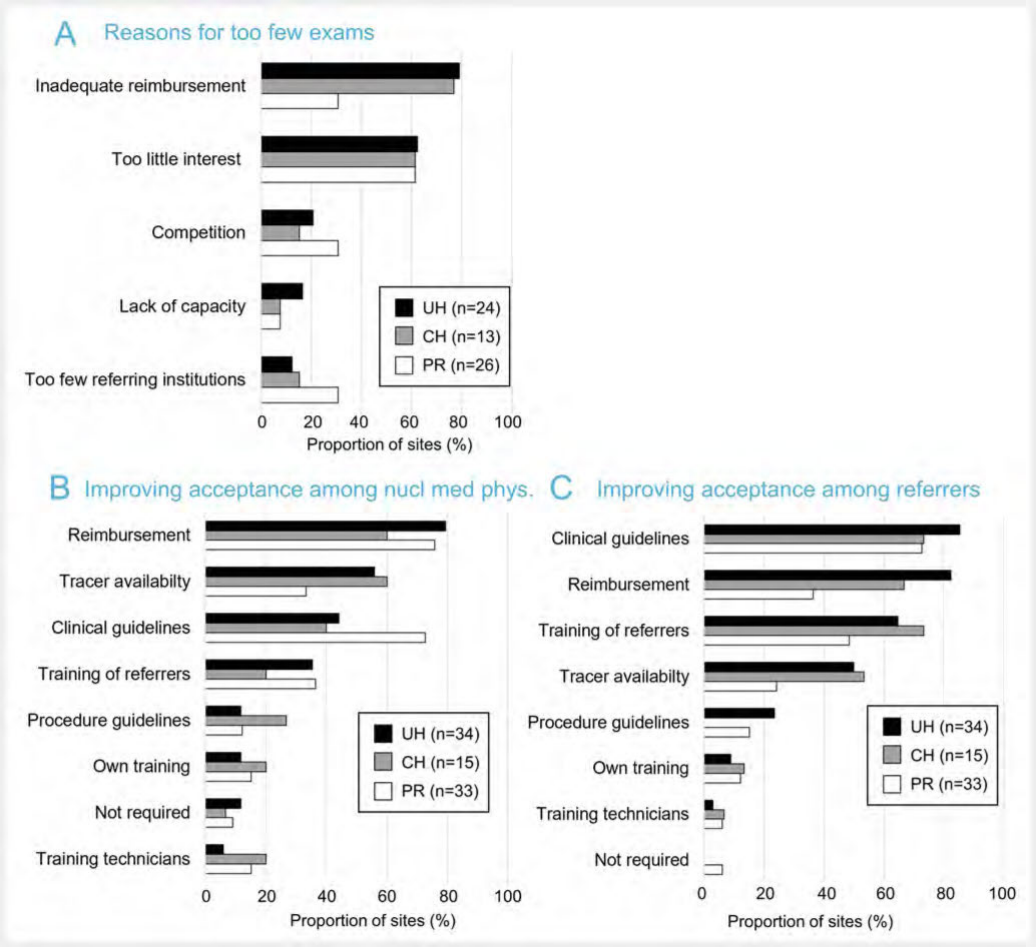
Assumed reasons for a too low number of neuroimaging procedures and measures to improve their acceptance. **A:** assumed reasons for “too few” (or “far too few”) nuclear neuroimaging procedures (“lack of or inadequate reimbursement”, “too little interest from referring physicians”, “competition from other nuclear medicine facilities”, “lack of capacity for nuclear neuroimaging procedures at the responding institution”, “too few referring institutions in the local area”; multiple answers possible), **B:** measures that would increase the willingness of the responding institutions to perform nuclear neuroimaging procedures (“better reimbursement”, “better tracer availability”, “stronger recommendation of nuclear neuroimaging procedures in the clinical guidelines of the referring medical societies”, “better training of referring physicians”, “better procedures guidelines (standardization) from nuclear medicine societies”, “better training of the nuclear medicine physicians at the responding institution”, “none/not required”, “better training of the nuclear medicine technicians at the responding institution”; multiple answers possible), **C:** measures that are expected to increase the acceptance of nuclear neuroimaging procedures by referring physicians (same response options as in B, but different ordering according to frequency; multiple answers possible). The number of responding institutions differed between the questions and therefore is given separately for each subplot. (PR = practice or medical supply center, CH = non-university community hospital, UH = university hospital).

Among those institutions reporting “too few” or “far too few” nuclear neuroimaging procedures ( Fig. 4 C), “inadequate reimbursement” was the most frequently cited reason for this ( Fig. 5 A). Consistently, “better reimbursement” was the most frequently selected measure to improve the acceptance of neuroimaging procedures by nuclear medicine facilities ( Fig. 5 B). Thus, better reimbursement has the greatest potential to increase the willingness of responding institutions to offer nuclear neuroimaging procedures. This is consistent with the fact that DAT SPECT, which is reimbursed by statutory health insurances, was performed by the vast majority of responding institutions ( Fig. 2 A). For CH and UH, we assume that the frequency of PET brain imaging procedures is limited by bureaucratic barriers such as internal billing and the additional effort for reimbursement applications for individual patients.

The lack of interest on the part of the referrers was the second most frequently cited reason for “too few” or “far too few” nuclear neuroimaging procedures ( Fig. 5 A). Better anchoring of the neuroimaging procedures in the clinical guidelines and specific training of the referring physicians were among the most frequently cited measures to remedy this ( Fig. 5 C). Concerning the anchoring in the clinical guidelines, the latest updates of the S3 guideline on dementia 4 and the S2k guideline on Parkinson’s disease 5 clearly strengthened the role of nuclear neuroimaging. However, further continuous effort is required to stabilize and/or further strengthen the role of nuclear neuroimaging in these guidelines. This is particularly true for “living” guidelines, such as the S3 guideline on dementia 4 , which are updated more frequently than “non-living” guidelines. Beyond clinical validity and utility, nationwide availability and sufficient capacity are important aspects for the anchoring of nuclear neuroimaging procedures in the clinical guidelines. It is therefore desirable that more nuclear medicine providers offer neuroimaging procedures, too, even if the number of examinations may be small initially. Concerning specific training on nuclear neuroimaging, about 60% of the responding nuclear medicine institutions considered “better training” of the referring physicians important ( Fig. 5 C), while only a minority (≤20%) considered “better training” of their own staff (physicians, technicians) important ( Fig. 5 B). This may be due to rater bias to some extent (overestimation of own expertise and underestimation of referrersʼ expertise). Nevertheless, it suggests that better training of referring physicians regarding nuclear neuroimaging procedures may be important to improve the status of nuclear neuroimaging in Germany. As a concrete measure, future editions of the training course on nuclear neuroimaging initiated in 2024 by the DGN working group on nuclear neuroimaging could be organised in close cooperation with the societies of referring physicians and aim for a balanced number of nuclear medicine specialists and referring physicians among the participants.

Up to about 25% of the responding institutions saw a need for improvement of the procedures guidelines for nuclear neuroimaging ( Fig. 5 B), probably with regard to standardisation of the procedures, more precise recommendations and inclusion of representative example images (→ training). Standardization of nuclear neuroimaging (e.g., according to EARL recommendations) has obvious advantages, for example in terms of comparability of semi-quantitative parameters between different sites, different cameras and follow-up examinations of the same patient. On the other hand, recommendations in practice guidelines should be broad enough (i) to allow all adequately equipped facilities to perform the procedures in accordance with the guidelines and (ii) to avoid inappropriate legal claims by patients.

Better availability of tracers was also considered important to improve acceptance of nuclear neuroimaging, particularly among CH and UH ( Fig. 5 B). We believe that this primarily relates to PET tracers, including (but not limited to) commercial amyloid tracers. The relatively low demand to date still affects the priority given by providers to the commercial supply of amyloid tracers. Some commercial providers offer their amyloid tracer in certain regions only, on selected working days, or at less favourable times (afternoon). In order to increase flexibility for users, further optimisation of tracer availability is desirable and can be expected in the future as demand increases.

### DAT SPECT methodology

**Fig. 6:**
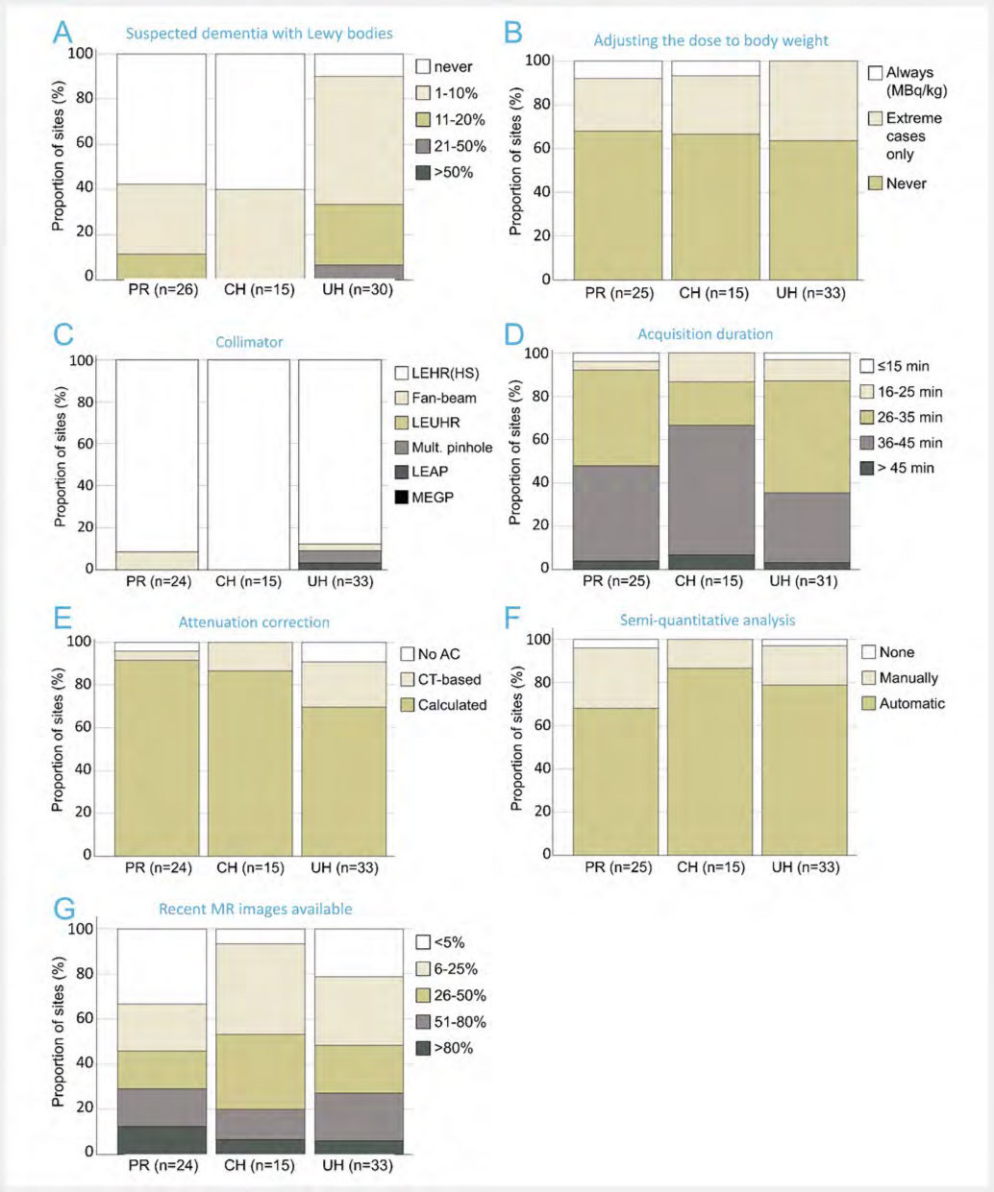
Dopamine transporter SPECT methods. **A:** relative frequency of “dementia with Lewy bodies versus dementia of the Alzheimer type” as indication, **B:** adjustment of the radioactivity dose in light-weight patients (< 70 kg), **C:** type of collimator used for the acquisition, **D:** total duration of the acquisition, **E:** use and type of attenuation correction, **F:** use and type of semi-quantitative (specific binding ratio) analysis, **G:** proportion of cases for which sufficiently up-to-date MR images (not only the written report) are available for the evaluation of the SPECT images. The number of responding institutions differed between the questions and therefore is given separately for each subplot. (PR = practice or medical supply center, CH = non-university community hospital, UH = university hospital).

Findings with reference to the DAT SPECT methodology are summarized in Fig. 6 .

While DAT SPECT was performed for the discrimination of dementia with Lewy bodies from AD at 90% of the UH performing DAT SPECT, about 60% of PR and CH performing DAT SPECT reported to never use it for this task ( Fig. 6 A). This is in line with the well-known underdiagnosis of dementia with Lewy bodies, particularly in primary and secondary care: dementia with Lewy bodies is diagnosed about 3 times more often in postmortem studies 20 than in clinical prevalence studies 21 .

Almost all (96%) of the responding institutions performing DAT SPECT used a fixed activity dose or adjusted (reduced) the dose only in extremely lightweight patients ( Fig. 6 B). However, a few (4%) of the institutions strictly adapted the activity dose according to the patients’ body weight, analogous to the linear activity dosing in brain FDG PET according to the current diagnostic reference levels (3 MBq/kg) published by the German Federal Office for Radiation Protection (Bundesamt für Strahlenschutz, BfS).

The vast majority of all responding institutions used a low-energy-high-resolution (LEHR) collimator for DAT SPECT (92%; Fig. 6 C) with scan duration between 25 and 45 minutes (85%; Fig. 6 D), in line with procedure guidelines for DAT SPECT 22 23 . Unexpectedly, fan-beam collimators were used at only 3 (4%) of all responding institutions (2 PR, 1 UH), although the DGN practice guideline for DAT SPECT states that “fan-beam collimators are generally preferable to parallel-hole collimators because they offer significantly higher count rates at the same resolution” 23 . The limited prevalence of fan-beam collimators may be explained by the additional costs for purchasing fan-beam collimators specifically for a limited number of brain scans and/or by the additional effort for changing collimators between consecutive patients. The latter may be minimized by “pooling” DAT SPECT examinations to scan them consecutively. Interestingly, two institutions reported acquisition duration of 15 minutes or less. One of these institutions used LEHR collimators. The other institution used brain-specific multiple-pinhole collimators, which provide greatly improved count sensitivity at similar (or better) spatial resolution even compared to fan-beam collimators 24 .

DAT SPECT was performed with correction for photon attenuation at the vast majority of institutions (94%), most often using calculated rather than CT-based attenuation correction (85% versus 15%, Fig. 6 E). Four institutions (6%) reported not to perform attenuation correction in DAT SPECT. This is in line with the EANM/SNMMI procedure guideline for dopaminergic imaging in parkinsonian syndromes that states that “corrections (attenuation, scatter and septal penetration, and partial volume effect) do not necessarily benefit visual interpretation” 22 . However, it is highly recommended to be strictly consistent with respect to the use of corrections (“always using the same methods or never”) 23 25 .

The vast majority of institutions reported to perform semi-quantitative analyses of DAT SPECT images (97%, Fig. 6 F), most of them using automatic methods rather than manual delineation of striatal regions-of-interest and the reference region (79% versus 21%). Two institutions (3%) reported not to perform semi-quantitative analyses. This is in line with the EANM/SNMMI procedure guideline stating that “visual interpretation of images is usually sufficient for clinical report in the majority of cases” 22 .

The proportion of cases for which sufficiently up-to-date MR images (not only the written report) are available for the evaluation of the DAT SPECT images was on average about one third ( Fig. 6 G), independent of the institution type (PR, CH, UH). This means that sufficiently up-to-date MR images are not available in about two thirds of the clinical DAT SPECT. The EANM/SNMMI procedure guideline states that “review of previous brain CT and MRI is mandatory” in order to avoid that structural lesions along the nigrostriatal pathway are misinterpreted as nigrostriatal degeneration 22 . The DGN DAT SPECT procedure guideline recommends to advise the patient to bring the results of any relevant previous examinations including CT and/or MR images of the brain 23 .

## Summary

The survey provided the first broad systematic assessment of the status of brain SPECT and PET in Germany. In practices, nuclear neuroimaging procedures are less frequently performed than in (community and university) hospitals and are often limited to dopamine transporter SPECT. Brain PET is mainly performed in hospitals, and in non-university community hospitals it is often restricted to FDG PET. Nevertheless, the availability of amyloid PET scans in Germany can be taken for granted on a large scale with well-certified quality. Access to amyloid PET will therefore not be a major bottleneck for new treatments of Alzheimer’s disease. Adequate reimbursement and clear anchoring in clinical guidelines have the greatest potential to advance nuclear neuroimaging in Germany. Dopamine transporter SPECT in clinical practice is largely in agreement with common procedure guidelines. A main area for improvement is the limited availability of sufficiently up-to-date MR images in order to avoid misinterpretation of structural/vascular lesions as indication of nigrostriatal degeneration.

## Supplementary Material

Supplementary Material Zusatzmaterial
