# Supplementary material for: The Status of Neuroimaging with SPECT and PET in Germany: Results from the 1 st Survey on Nuclear Neuroimaging in Germany in 2023
Source: Nuklearmedizin. 2025 Apr 15;64(4):250–61. doi: 10.1055/a-2566-1487 (PMC12328034; doi:10.1055/a-2566-1487)
Supplement: Supplementary file 1 — Supplementary Material [file 10-1055-a-2566-1487_25662714.pdf]

Supplementary Material

The Status of Neuroimaging with SPECT and PET in Germany: Results from the 1st Survey on Nuclear Neuroimaging in Germany in 2023

Ralph Buchert[1,*], Alexander Drzezga[2,3,4,*], Mathias Schreckenberger[5], Karl-Josef Langen[6,7,8] and Philipp T. Meyer[9] , for the Working Group Nuclear Brain Imaging of the German Society of Nuclear Medicine (DGN e.V.)

[1]Department of Diagnostic and Interventional Radiology and Nuclear Medicine, University Medical Center Hamburg-Eppendorf, Hamburg, Germany

[2]Institute of Neuroscience and Medicine (INM-2), Forschungszentrum Jülich, Germany

[3]Department of Nuclear Medicine, Faculty of Medicine and University Hospital Cologne, University of Cologne, Germany

[4]German Center for Neurodegenerative Diseases (DZNE), Bonn-Cologne, Germany

[5]Department of Nuclear Medicine, Johannes Gutenberg University, 55101 Mainz, Germany

[6]Institute of Neuroscience and Medicine (INM-3/INM-4/INM-5/INM-11), Forschungszentrum Jülich, Jülich, Germany

[7]Department of Nuclear Medicine, University Hospital RWTH Aachen, Aachen, Germany

[8]Center of Integrated Oncology, Aachen Bonn Cologne Düsseldorf, Germany.

[9]Department of Nuclear Medicine, Medical Center - University of Freiburg, Freiburg, Germany

[*]These authors contributed equally as first authors

Nuklearmedizin | DOI 10.1055/a-2566-1487 | © Thieme. All rights reserved.

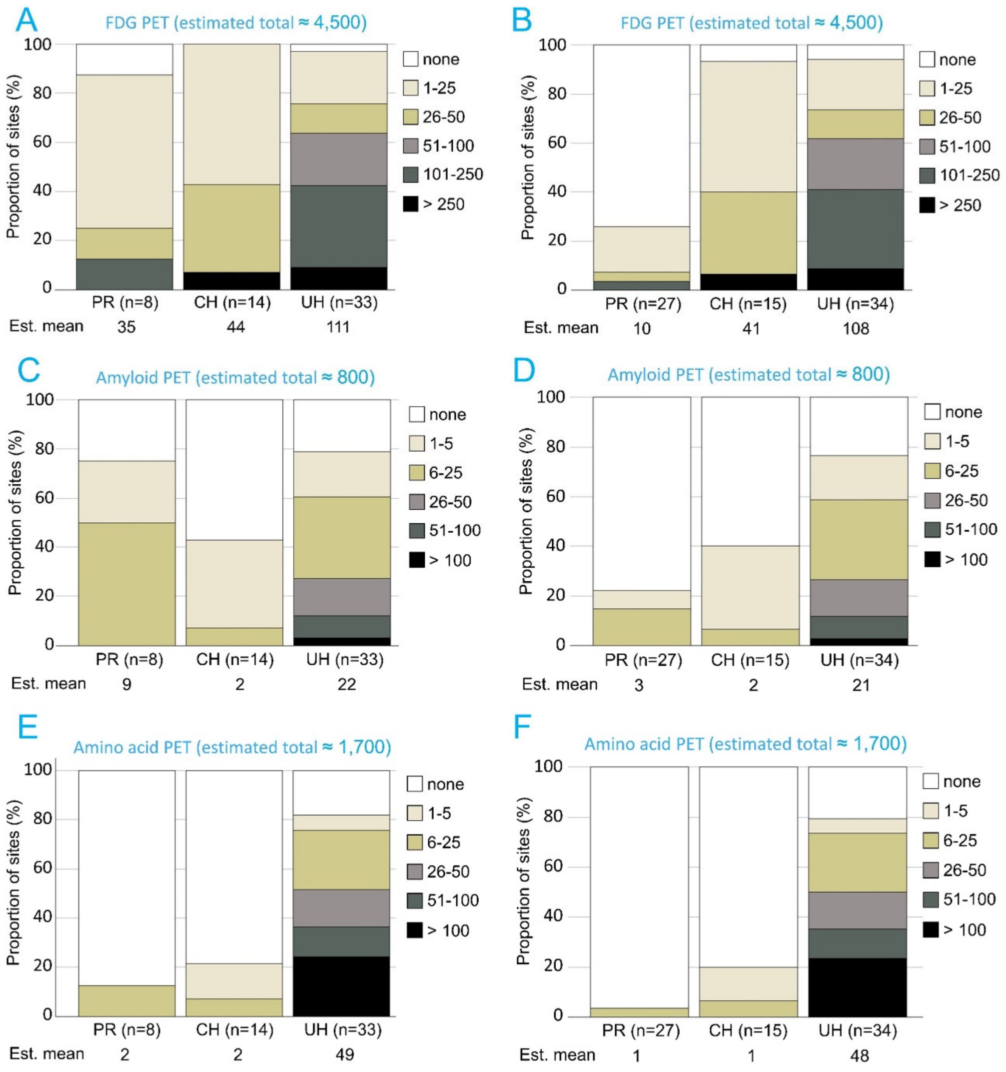

Supplementary Figure 1 Annual number of FDG PET (A, B), amyloid PET (C, D) and amino acid PET (E, F) scans. In the subplots of the left column (A, C, E), percentages were computed relative to the institutions with a PET scanner available (8 PR, 14 CH, 33 UH). In the corresponding subplots of the right column (B, D, F), percentages were computed relative to all institutions performing any nuclear neuroimaging procedures independent of PET availability (27 PR, 15 CH, 34 UH).

Nuklearmedizin | DOI 10.1055/a-2566-1487 | © Thieme. All rights reserved.

German federal states	Number of trained physicians
Baden-Württemberg	44
Bayern	94
Berlin/Brandenburg	64
Bremen	3
Hamburg	10
Hessen	23
Mecklenburg-Vorpommern	11
Niedersachsen	36
Nordrhein-Westfalen	115
Rheinland-Pfalz	16
Saarland	22
Sachsen	10
Sachsen-Anhalt	16
Schleswig-Holstein	12
Thüringen	7
Total	483

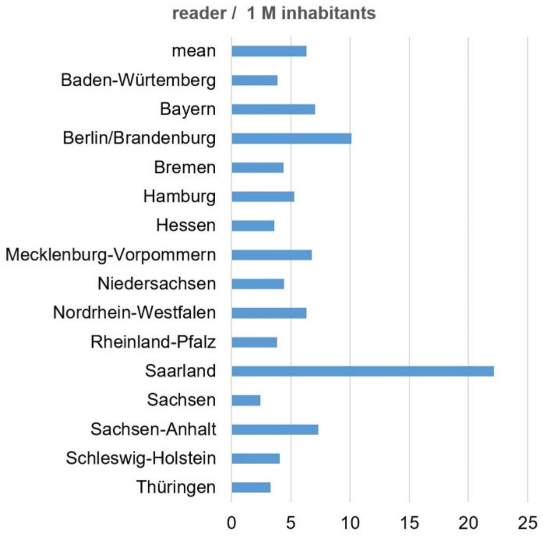

Supplementary Figure 2 Number of physicians trained for the reading of amyloid PET scans with [^{18}F]florbetaben or [^{18}F]flutemetamol. Physicians trained for both tracers were counted twice. (Sources: number of trained physicians: German Electrical and Electronic Manufacturers' Association ZVEI, 03/2024; population: Destatis, 12/2022).

Nuklearmedizin | DOI 10.1055/a-2566-1487 | © Thieme. All rights reserved.

Supplementary Tab. 1 Number of responding institutions across the German federal states (population: Destatis, 12/2022). (PR = practice or medical supply center, CH = non-university community hospital, UH = university hospital)

German federal state	PR	CH	UH	Total (n)	Total per 1 M habitants
Baden-Württemberg	4	2	5	11	0.98
Bayern	7	2	5	14	1.05
Berlin/Brandenburg	0	2	1	3	0.47
Bremen	1	0	0	1	1.46
Hamburg	2	0	1	3	1.59
Hessen	2	0	2	4	0.63
Mecklenburg-Vorpommern	0	0	2	2	1.23
Niedersachsen	2	1	2	5	0.61
Nordrhein-Westfalen	10	5	9	24	1.32
Rheinland-Pfalz	2	1	1	4	0.96
Saarland	0	0	0	0	0.00
Sachsen	3	2	2	7	1.71
Sachsen-Anhalt	0	0	1	1	0.46
Schleswig-Holstein	0	0	2	2	0.68
Thüringen	0	0	1	1	0.47
Total	33	15	34	82	0.97

Nuklearmedizin | DOI 10.1055/a-2566-1487 | © Thieme. All rights reserved.
